# Association of Homozygous Thrombophilia Polymorphisms and Venous Thromboembolism in Shahrekord, Iran

**Published:** 2016

**Authors:** Hamid Rouhi-Broujeni, Batoul Pourgheysari, Ali-Mohammad Hasheminia

**Affiliations:** 1 Clinical Biochemistry Research Centre, Shahrekord University of Medical Sciences, Shahrekord, Iran; 2 Paramedical School and Cellular and Molecular Research Centre, Shahrekord University of Medical Sciences, Shahrekord, Iran; 3 School of Nursing and Midwifery, Shahrekord University of Medical Sciences, Shahrekord, Iran

**Keywords:** Venous thromboembolism, Factor V Leiden, methylenetetrahydrofolate reductase, Homozygous, FIIG20210A polymorphism

## Abstract

**Background:**

Venous thromboembolism (VTE) is a major cause of mortality. Factor V Leiden (FVL), methylenetetrahydrofolate reductase (MTHFR) C677T, and prothrombin (FII) G20210A polymorphisms are the main inherited risk factors for VTE. Since evidence is limited on homozygotes, the aim of this study was to investigate the association between homozygous variants of these polymorphisms and VTE in Shahrekord, southwest Iran.

**Materials and Methods:**

In this case-control study, blood samples of 72 VTE patients admitted to Hajar Hospital, Shahrekord and 306 sex- and age-matched healthy volunteers as controls were taken in EDTA Vacutainers. The polymorphisms of FVL, MTHFR C677T, and FIIG20210A were investigated by PCR-RFLP. The data were analyzed by descriptive statistics and independent t-test.

**Results:**

The frequency of all homozygous polymorphisms was found to be 16.77% in patients and 4.90% in controls with a significant difference (P=0.004). Homozygous FVL mutation was more frequent in patients than in controls with no significant difference. Regarding the frequency of homozygous MTHFR C677T, a significant difference was noted between patients and controls (P=0.03). There was no significant difference in homozygous FIIG20210A and heterozygous variants of the above-mentioned polymorphisms between the patients and controls.

**Conclusion:**

Homozygous MTHFR C677T polymorphism is associated with VTE in Shahrekord. Control of the acquired risk factors may be necessary in homozygous form of this polymorphism. VTE patients with this polymorphism may need to be managed differently.

## INTRODUCTION

Venous thromboembolism is a disease in which both inherited and acquired factors may be involved. Although the real prevalence of VTE, consisting of deep venous thrombosis (DVT) and pulmonary embolism (PE), remains to be determined in many communities, it is known to be one of the main causes of mortality. In the United States, VTE is considered as the third leading cause of mortality and a leading cause of unpredictable deaths (1). Even the people who survive PE have a lower quality of life than healthy people (2).

Coagulation factor deficiencies, platelets abnormalities, impaired vessel wall, and coagulation inhibitors can contribute to developing thrombosis. Thrombophilia is a condition which is conducive to developing thrombosis. Factor V Leiden (FVL), prothrombin (FII) G20210A, and methyltetrahydrofolate reductase (MTHFR) C677T polymorphisms are the main risk factors for VTE. Moreover, the role of factor XIII and platelet glycoprotein IIb/IIIa has been recently considered in VTE (3, 4). FVL is a mutation of coagulation factor V that makes FVL resistant to protein C, its inhibitor (5). MTHFR plays an important role in maintaining homocysteine level. The C677T polymorphism of MTHFR molecule makes the enzyme heat resistant and may increase serum homocysteine which is a predisposing factor for thrombosis (6). The MTHFR C677T has been reported to be associated with increased VTE in a Chinese population but some conflicting data have been reported in other ethnicities (7). The FIIG20210A polymorphism is associated with increased plasma prothrombin level and increased risk of venous thrombosis (8), and the FIIG20210A carriers are at 2–3 times higher risk for developing VTE (9, 10). The frequencies of these polymorphisms vary depending on the racial origins in both normal and VTE patient populations (9–11), and the available data confirm widely variable distribution of different polymorphisms.

We have already found that PLA2 polymorphism of platelet glycoprotein IIb-IIIa is associated with increased risk of developing VTE and severity of PE (3, 12). Because homozygosity in any of the polymorphisms can increase the gene products and relevant data are limited, the aim of this study was to investigate the association between homozygous variants of these mutations and VTE.

## MATERIALS AND METHODS

### Patients

In this study, 72 patients with VTE who were admitted to Hajar Hospital, Shahrekord, Iran and presented with one or more symptoms of PE or DVT at the time of diagnosis were enrolled. The control group consisted of 306 sex and age-matched healthy individuals without history of venous or arterial diseases living in the same region. Both patients and healthy controls provided informed consent to participate in the study before blood sample collection. The ethical approval for the study was obtained from the Shahrekord University of Medical Sciences Ethics Committee (Code; 638).

### Detection of polymorphisms

All the patients and controls were investigated for FVL, MTHFR and FIIG20210A polymorphisms. Blood samples were taken from the participants in EDTA Vacutainers and transferred to the Cellular and Molecular Research Center of the Shahrekord University of Medical Sciences. DNA was extracted from total blood by phenyl-chloroform extraction, quantitated by a spectrophotometer (Unico 2010), and then standardized. The polymorphisms of interests were investigated by PCR-RFLP. A DNA-free solution was used as negative control.

The genes of interest were amplified by a thermocycler (ASTEC, PC818, Japan). The primers which were used to investigate each mutation were designed according to Ivanov et al. study (13), and are listed in Table 1. The restriction enzymes consisted of Mn*1l* for FVL, Hin*f1* for MTHFR C677T, and Hin*dlll* for FIIG20210A. The restriction enzymes were provided by Tags (Copenhagen, Denmark) and the primers were purchased from Fermentase (Russia). An 8% polyacrylamide gel was used to investigate the final products and the bands were observed by Coomassie Brilliant Blue staining.

**Table 1.** Primer sequences of genetic polymorphisms

**Genetic polymorphisms**	**Primers**
FVL	F 5′ TGC CCA GTG CTT AAC AAG ACC A 3′
	R 5′ TGT TAT CAC ACT GGT GCT AA 3′
MTHFR C677T	F 5′ TGA AGG AGA AGG TGT CTG CGG GA 3′
	R 5′ AGG ACG GTG CGG TGA GAG TG 3′
FIIG20210A	F 5′ TCT AGA AAC AGT TGC CTG GC 3′
	R 5′ ATA GCA CTG GGA GCA TTG AAG C 3′

The thermal cycling programs of the studied polymorphisms were as follows:

FVL mutation: Four minutes at 96°C, six cycles of 60 seconds at 96°C, 60 seconds at 58°C, and 60 seconds at 72°C, followed by 28 cycles of 30 seconds at 96°C, 30 seconds at 56°C, and 30 seconds at 72°C, and a final extension of 5 minutes at 72°C.

MTHFR C677T polymorphism: Five minutes at 94°C followed by five cycles of 60 seconds at 94°C, 60 seconds at 59°C, and 60 seconds at 72°C, and then 25 cycles of 30 seconds at 94°C, 30 seconds at 59°C, and 30 seconds at 72°C, and a final extension of 10 minutes at 72°C.

FIIG20210A polymorphism: Five minutes at 94°C followed by 33 cycles of 30 seconds at 94°C, 45 seconds at 60°C, and 60 seconds at 72°C, and a final extension of 5 minutes at 72°C.

A 267-bp fragment was amplified from FVL by the primers required to identify G1691A mutation. In the un-mutated allele, two cleavage sites of the Mn*1l* enzyme are present and therefore three fragments of 163, 67, and 37 bp are developed. If the mutation of interest exists, an enzyme site is deleted via G --> A at the point 1691 of the relevant gene and two fragments of 200 and 67 bp are developed.

Using the primers required to identify the MTHFR C677T polymorphism, a 198-bp fragment was amplified. In the un-mutated allele, there is no cleavage site of Hin*fl* enzyme and therefore the 198-bp fragment remains. If the mutation of interest exists, an enzyme site is developed in the 198-bp fragment through C --> T at the point 677 of the relevant gene and two fragments of 175 and 23 bp are developed.

To detect the FIIG20210A polymorphism, a 345-bp fragment was amplified. The wild variant of this polymorphism has no cleavage site of Hin*dIII* enzyme. If the FIIG20210A polymorphism is present, a cleavage site is created for the enzyme and two fragments of 322 and 23 bp are developed.

### Statistical analysis

The data were analyzed by SPSS 18. Descriptive statistics were used for the demographic characteristics. The significance of the differences in the distribution of homozygous polymorphisms between the patients and the controls was investigated by chi-square test, and P<0.05 was considered significant. Moreover, the odds ratio (OR) was used to represent the association of the polymorphisms with VTE.

## RESULTS

### Demographic characteristics of patients

Thirty-seven patients had PE and the remaining had DVT. The mean age of the patients was 42.12±18.59 years; 40.27% of the patients were males. The mean age of male patients was lower than that of female patients with no significant difference (P>0.05). There was no significant difference in the age of patients with homozygous and heterozygous mutations.

### Homozygous and heterozygous polymorphisms in patients

**Figure 1.**
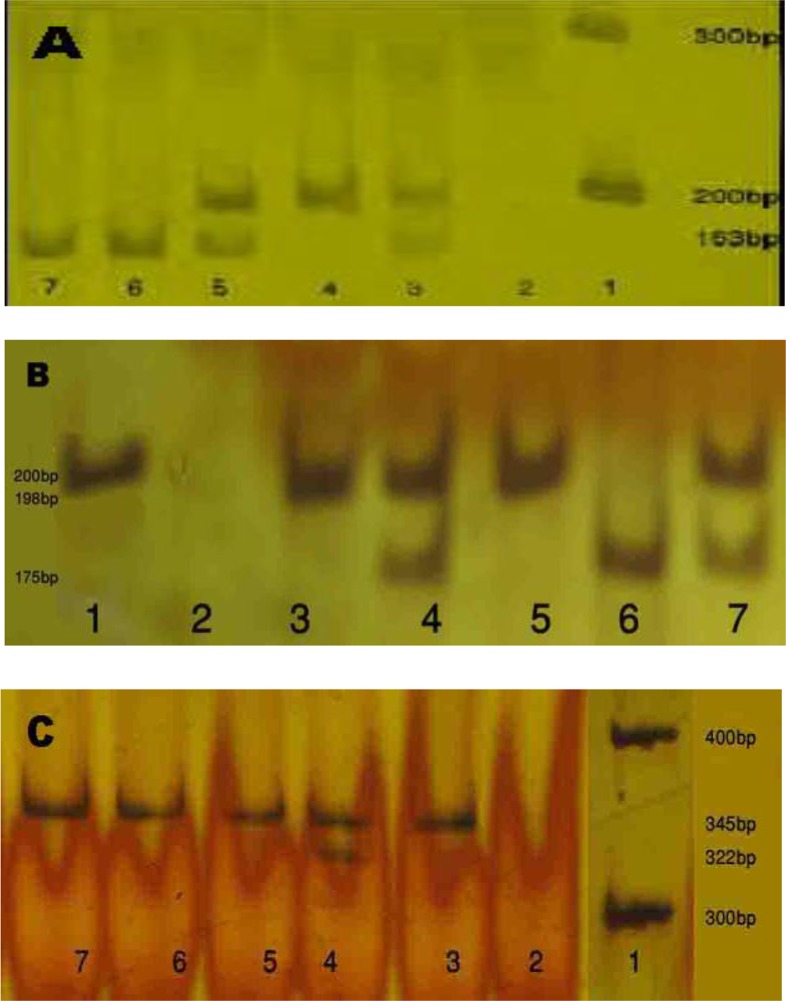
PCR-RFLP products of investigated polymorphisms on 8% polyacrylamide gel. A) FVL polymorphism: 1 indicates DNA marker, 4 homozygous FVL mutation, 3 & 5 heterozygous and 6 & 7 wild type. B) MTHFR C677T polymorphism: 1 indicates DNA marker, 6 homozygous mutation, 4 & 7 heterozygous and 3 & 5 wild type. C) FIIG20210A: 1 indicates DNA marker, 4 heterozygous and other bands wild type.

Out of 72 studied patients, 16.66% and out of controls, 4.90% had homozygous mutations of the studied polymorphisms (P=0.004, Table 2). FVL was seen in four (5.55%) patients, MTHFR C677T in 32 (44.14%), and FIIG20210A in one (1.4%) patient. Table 3 compares the frequency of heterozygous and homozygous polymorphisms between the patients and controls. Figure 1 illustrates the PCR-RFLP products of FVL, MTHFR C677T, and FIIG20210A on polyacrylamide gel.

**Table 2.** Comparison of homozygous thrombophilia polymorphisms between VTE patients and controls

	**Frequency**	**Percentage**	**Mean polymorphism/individual**	**P-value**
**Patients (72)**	12	16.66	0.2	0.004
**Controls (306)**	15	4.90	0.04	

**Table 3.** Comparison of homozygous and heterozygous thrombophilia polymorphisms between VTE patients and controls

	**Heterozygote polymorphisms**		**Homozygote polymorphisms**	
	
**Polymorphisms**	**Patients**	**Controls**	**Patients**	**Controls**
FVL number (%)	2 (2.77)	6 (1.95)	2 (2.77)	1 (0.33)
OR (95% confidence interval)	1.42(0.28–0.23)		8.7 (0.78–7.46)	
P value	NS^*^		0.09	
MTHFRC677T number (%)	24 (33.33)	98(32.03)	8 (11.11)	12 (3.92)
OR (95% confidence interval)	1.06 (0.62–1.83)		3.06 (1.23–0.84)		
P value	NS		0.03		
FIIG20210A Number (%)	1 (1.4)	3 (1)	0	0
OR (95% confidence interval)	1.42 (0.146–13.88)		-	
P value	NS		-	

FVL: Factor V Leiden, MTHFRC677T: Polymorphism C677T in methylenetetrahydrofolate reductase, FII: Prothrombin

NS: Not significant

Homozygous FVL mutation had a frequency of 2.77% in the patients and 0.32% in the controls with no significant difference (P=0.09); 11.11% of the patients vs. 3.92% of the controls were homozygous for MTHFR C677T (OR=3.06, 95% CI: 1.32–7.48). No significant difference was noted in the heterozygous MTHFR C677T between the patients and the controls (P>0.05). No patients or controls were seen to be homozygous for FIIG20210A polymorphism. The frequencies of heterozygous MTHFR C677T and FIIG20210A polymorphisms were not significantly different between the patients and the controls (Table 3).

In two patients, one PE and one DVT, (and none of the controls) double homozygous FVL and MTHFR C677T was seen. No cases of triple homozygosity were found in the patients or the controls.

## DISCUSSION

This study demonstrated that VTE was associated with a number of homozygous thrombophilic polymorphisms, and some of the known thrombophilic homozygous polymorphisms increased the risk of developing VTE, while others had no effect. In a study on factor XIII polymorphisms, homozygous FXIIIA-V34L was found to protect against VTE, while heterozygous FXIIIA-V34L and XIIIB-H95R had no effect (4). Besides, the frequency of coincidence of thrombophilic mutations was found to be higher in DVT patients than in PE patients and controls (14). In a study conducted in an Australian Caucasian population, 64.2% and 24.5% of studied group were homozygous and heterozygous, respectively at least for one of the inherited thrombophilia; these values are higher than our findings (15). Among the patients in our study, the most frequent homozygous polymorphism was found to be MTHFR C677T (11.11%). This polymorphism is distributed among all populations and is more frequent in some racial populations (9, 10). It has been found more commonly in Australians, Europeans and Chinese than African-Americans (15–17). Gohil et al. reported a significant association between MTHFR C677T and DVT in Chinese/Thai population but not in Caucasians (9).

The frequency of homozygous MTHFR C677T was 3.9% in the controls in our study, which was relatively lower than the values reported from Europe and Australia (15, 18). The significantly higher frequency of homozygous MTHFR C677T in patients than controls in our study is inconsistent with the results of Ivanov et al, reporting a higher frequency in the control group (13). In carriers of heterozygous mutation, enzyme function is about 65% of its normal level, whereas, this rate is only 30% in homozygous mutation (6). The presence of this polymorphism causes an increase in plasma homocysteine level that can contribute to the pathogenesis of arterial and venous thrombosis (19, 20). Kokturk et al. reported increased plasma homocysteine levels in patients, such that the risk of developing VTE increased by 4.8 times (20). In their study, the prevalence of homocysteinemia in VTE was 63% and higher in PE patients compared to DVT. Moreover, increased homocysteine can be due to systemic or endothelial oxidative stress which leads to platelet activation (21).

Homozygous FVL was more frequent in patients than in controls (2.77% vs. 0.32%) in our study, yet with no significant difference. Different studies have investigated the contribution of FVL mutation to VTE and reported various frequencies of this mutation in different populations (9, 22, 23). Coppola et al. found homozygous FVL to be associated with a higher risk of thrombosis while heterozygous FVL was associated with a lower risk of thrombosis (24). In a study in Turkey, homozygous FVL was significantly associated with thrombosis; whereas, MTHFR C677T was equally distributed in patients and controls (25), but in a Chinese population, FVL mutation was not associated with VTE, which is in agreement with our findings (9). The inconsistent findings of different studies can be explained by the racial origins and also the environmental risk factors influencing the whole population of a community, including people without these polymorphisms, and making them predisposed to thrombosis.

FIIG20210A mutation was not associated with VTE in our study which is consistent with the results of Bezgin et al, who found similar frequencies of this mutation in patients with different manifestations of VTE and controls (26). Some studies have reported the association of FIIG20210A mutation with VTE pathogenesis (8), while some others have found such associations in Caucasians but not in Chinese/Thai or African American populations (9). The latter is in agreement with our study.

Coinheritance of thrombophilic factors can reduce the predisposition to bleeding in hemophilia patients (27) but increase the risk of thrombosis in VTE patients (3). We had two patients but no controls with coinheritance of homozygous FVL and MTHFR C677T in this study. Because of the limited number of patients in our study, we could not investigate the significance of such coinheritance. We studied a limited number of homozygous thrombophilia polymorphisms, but some other polymorphisms may also be involved in VTE, which can be investigated in future studies.

## CONCLUSION

This study demonstrated that 16.6% of VTE patients versus 4.90% of controls had thrombophilia homozygous polymorphisms and the homozygous MTHFR C677T polymorphism was associated with increased risk of developing thrombosis in patients with VTE in Shahrekord. These patients may need to be managed differently. Knowledge about the presence of these risk factors can help to prevent these diseases, especially among patients’ relatives who are carriers of these polymorphisms, and control the acquired risk factors.
